# The impact of male factors and their correct and early diagnosis in the infertile couple's pathway: 2021 perspectives

**DOI:** 10.1007/s40618-022-01778-7

**Published:** 2022-03-29

**Authors:** F. Pallotti, A. Barbonetti, G. Rastrelli, D. Santi, G. Corona, F. Lombardo

**Affiliations:** 1 Laboratory of Seminology-Sperm Bank “Loredana Gandini”, Department of Experimental Medicine, “Sapienza” University of Rome, Viale del Policlinico 155, 00161 Rome, Italy; 2 Andrology Unit, Department of Life, Health and Environmental Sciences, University of L’Aquila, L’Aquila, Italy; 3 Andrology, Women’s Endocrinology and Gender Incongruence Unit, Careggi Hospital-Department of Experimental, Clinical and Biomedical Sciences, University of Florence, Florence, Italy; 4 Unit of Endocrinology, Department of Biomedical, Metabolic and Neural Sciences, University of Modena and Reggio Emilia, Modena, Italy; 5 Endocrinology Unit, Medical Department, Maggiore-Bellaria Hospital, Azienda-Usl Bologna, 40139 Bologna, Italy

**Keywords:** Couple infertility, Male factor, Semen analysis, FSH, ART

## Abstract

**Purpose:**

The current clinical practice in reproductive medicine should pose the couple at the centre of the diagnostic–therapeutic management of infertility and requires intense collaboration between the andrologist, the gynaecologist and the embryologist. The andrologist, in particular, to adequately support the infertile couple, must undertake important biological, psychological, economical and ethical task. Thus, this paper aims to provide a comprehensive overview of the multifaceted role of the andrologist in the study of male factor infertility.

**Methods:**

A comprehensive Medline, Embase and Cochrane search was performed including publications between 1969 and 2021.

**Results:**

Available evidence indicates that a careful medical history and physical examination, followed by semen analysis, always represent the basic starting points of the diagnostic work up in male partner of an infertile couple. Regarding treatment, gonadotropins are an effective treatment in case of hypogonadotropic hypogonadism and FSH may be used in men with idiopathic infertility, while evidence supporting other hormonal and nonhormonal treatments is either limited or conflicting. In the future, pharmacogenomics of FSHR and FSHB as well as innovative compounds may be considered to develop new therapeutic strategies in the management of infertility.

**Conclusion:**

To provide a high-level of care, the andrologist must face several critical diagnostical and therapeutical steps. Even though ART may be the final and decisive stage of this decisional network, neglecting to treat the male partner may ultimately increase the risks of negative outcome, as well as costs and psychological burden for the couple itself.

## Introduction

Human reproduction has always been a topic of great interest and concern. An overwhelming amount of knowledge on pathophysiology of reproduction has been published since the spread of assisted reproduction techniques (ART) for both female and male factor infertility (tubal obstruction, oligo-astheno-teratozooospermia, etc.). The introduction of intra-cytoplasmic sperm injection (ICSI) constituted a major impulse for andrology, forcing researchers to deepen the knowledge on sperm fertilising ability and related functional and genetic problems [1]. The current clinical practice should pose the couple at the centre of the diagnostic–therapeutic management of infertility. For this path to be clear and fast, an intense collaboration is needed among the medical personnel assisting the couple, in particular between the andrologist, the gynaecologist and the embryologist. Nonetheless, it should be stressed that the possibility of directing the couple to ART to quickly respond to the couple’s needs (especially in case of an advanced maternal age) should not lead the reproductive health specialists into the temptation to overlook investigating thoroughly the causes of infertility. Neglecting to treat the couple, in fact, may ultimately increase the risks of negative outcome of the ART treatment [2], as well as costs and psychological burden for the couple itself. Also, it has been demonstrated that poor semen quality is an independent biomarker of poor general health, irrespective of detectable hypogonadism, allowing the andrologist to offer the patient a timely and precautionary diagnostic workup of any clinically important comorbidity [3].

From the point of view of the andrologist, the widespread availability of ART poses him/her in the middle of a complex decisional network. Although the role of the andrologist in the couple infertility is mainly clinical, he/she must undertake serious biological, psychological, economical and ethical tasks [1]. Benefits and costs of the diagnostic and therapeutic work up, also measured as time needed to achieve the desired result, must be balanced in order to adequately support the infertile couple. Thus, the aim of this paper is to provide the reader a comprehensive overview of the multifaceted role of the andrologist in the study of male factor infertility, the correct and early diagnosis, and the future perspectives in the infertile couple's work up.

## Methods

A comprehensive Medline, Embase and Cochrane search was performed including the following words: ("couple"[All Fields] OR "couples"[All Fields]) AND ("infertility"[MeSH Terms] OR "infertility"[All Fields] OR "infertile"[All Fields]) AND ("diagnosis"[MeSH Terms] OR "diagnosis"[All Fields] OR "diagnoses"[All Fields] OR "diagnosing"[All Fields] OR "diagnosis"[MeSH Subheading] OR ("workup"[All Fields] OR "workups"[All Fields]) OR ("semen analysis"[MeSH Terms] OR "semen analysis"[All Fields]) OR ("spermatozoa"[MeSH Terms] OR "spermatozoa"[All Fields] OR "sperm"[All Fields]) AND ("dna fragmentation"[MeSH Terms] OR ("dna"[All Fields] AND "fragmentation"[All Fields]) OR "dna fragmentation"[All Fields]))) AND ((("fertility"[MeSH Terms] OR "fertility"[All Fields] OR "fertile"[All Fields]) AND ("therapeutics"[MeSH Terms] OR "therapeutics"[All Fields] OR "treatments"[All Fields] OR "therapy"[MeSH Subheading] OR "therapy"[All Fields] OR "treatment"[All Fields] OR "treatment s"[All Fields])) OR "fsh"[All Fields]). Publications between 1969 and 2021 were included. When available, meta-analytic data were preferred. Further articles were retrieved from the papers’ reference lists.

## Diagnostic work up of men from the infertile couple

**Fig. 1:**
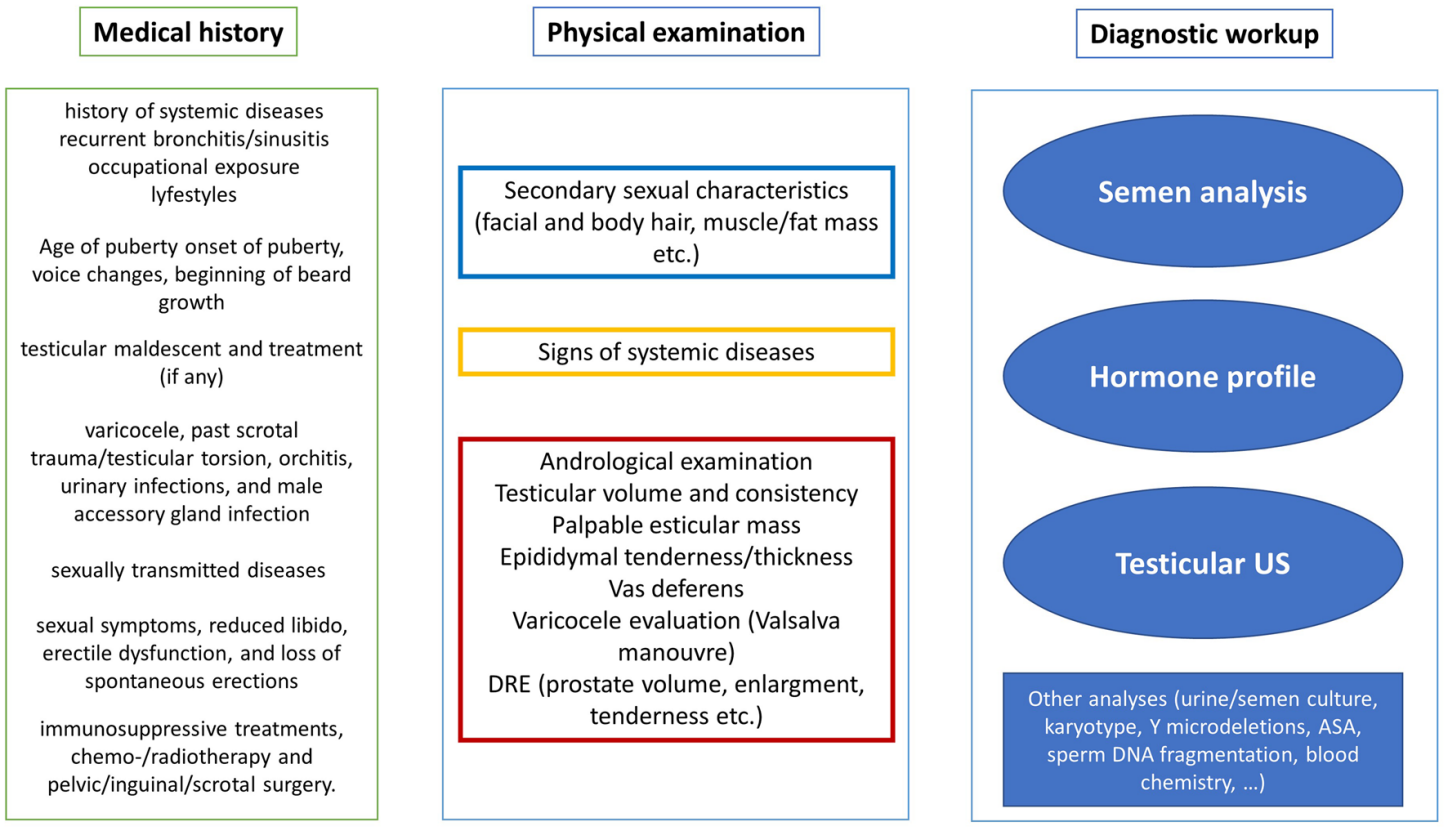
Summary of the andrological work up of the male partner from an infertile couple

Careful medical history and physical examination, followed by semen analysis, represent the basic starting points of the diagnostic work up in male partner of an infertile couple (Fig. 1).

Information collected during *medical history* can orient towards possible infertility aetiologies and risk factors. Familiar medical history, including data on the fertility status of parents and siblings, could raise suspicion of genetic causes of hypogonadism and infertility. The onset of puberty, voice changes and the beginning of beard growth must be recorded. Valuable data during medical history should consider information about any testicular maldescent and the age at which treatments (medical therapy or orchidopexy) were carried out [4], history of systemic diseases, varicocele, past-scrotal trauma and testicular torsion, orchitis (e.g. orchitis by mumps), urinary infections, sexually transmitted diseases, and urogenital infections, including prostatitis, vesiculitis, and epididymitis [5–7]. Recurrent bronchitis or sinusitis in childhood or adulthood could suggest specific disorders of respiratory system associated with infertility (e.g. ciliary dyskinesia, Kartagener syndrome, Young syndrome, or cystic fibrosis). Iatrogenic factors to be considered include immunosuppressive treatments, chemo-/radiotherapy and pelvic/inguinal/scrotal surgery. Occupational exposure to toxicants and physical agents, as well as lifestyles (e.g. anabolic substance abuse, tabagism, alcohol, diet), should also be carefully investigated [8]. Moreover, fever during the previous three months must be ruled out because of its possible impact on semen quality. Finally, sexual symptoms, such as decreased sexual desire and fantasies, erectile dysfunction, and loss of spontaneous nocturnal and morning erections, could suggest an androgen deficiency syndrome [9, 10].

At the *physical examination*, clinician should pay attention to the general signs of hypoandrogenization, as well as abnormalities in scrotal content. As seminiferous tubules largely account for the total testis volume which, in turn, correlates with the sperm output [11], the clinical assessment of testis volume (by Prader orchidometer) and consistency can provide a rough indication of spermatogenetic efficiency of the testis. Physical examination of the testis is also of utmost importance for the screening of testicular cancer. Valuable information also arises from palpatory assessment of proximal seminal tract: an enlarged epididymis might orient towards an obstructive disorder, while the absence of vas deferens might suggest their agenesis. Thickened or tender epididymis and tender vas deferens might result from inflammatory processes. Manual evaluation of pampiniform venous plexus of the standing patient, both at rest and after Valsalva manoeuvre, allows the clinical diagnosis and grading of varicocele which will be fully characterized through colour Doppler ultrasound (US). Additional information can be provided by digital rectal examination (DRE): a small prostate volume can reflect an androgen deficiency, an overall enlargement would suggest benign prostatic hyperplasia (BPH), while a knobby prostate surface with hard consistency can reflect the presence of a carcinoma. In the presence of prostatitis, the gland is painful and displays a doughy, soft consistency at the DRE; leukocytes can be detected in prostate fluid after prostatic massage [10, 12].

*Semen analysis*, performed in specialized laboratories by trained and experienced personnel, according to the latest WHO recommendations [13], represents the first level laboratory investigation in the diagnostic work up of men from the infertile couple. Semen analysis allows to recognize two main causes of male factor infertility which are very different from a prognostic point of view: azoospermia, leading to male sterility due to absolute inability to conceive, and oligo-astheno-teratozoospermia (OAT), where natural conception, albeit unlikely in many cases, can be still possible, especially in the presence of a high female fertility potential [14]. Computer-aided semen analysis (CASA) is currently used is some laboratories to perform semen analyses. It should be stressed however, that CASA systems are best used for the kinematic analysis and their utilization require extensive training [13]. Furthermore, being CASA an advanced examination, more suited for research settings, it should not be routinely used for the initial evaluation of the infertile male.

### Diagnostic work up of azoospermia

Azoospermia, defined as the absence of spermatozoa in the ejaculate, must be confirmed after the centrifugation of the semen sample and a thoroughly examination of the pellet. If spermatozoa are absent from fresh sample but observed in a centrifuged pellet, a cryptozoospermia can be diagnosed.

The main differential diagnosis in azoospermic patients in terms of treatment and prognosis is between testicular failure (nonobstructive azoospermia, NOA) and obstruction of the male reproductive tract (obstructive azoospermia, OA). Information about testicular volume along with basal serum levels of follicular stimulating hormone (FSH), luteinizing hormone (LH), and testosterone in the morning, can drive the diagnosis, identifying an underlying hypogonadism [15–17]. The evaluation of SHBG should also be considered to calculate free testosterone [18].

- Low testosterone combined with low/inadequately normal gonadotropin levels identifies a hypogonadotropic hypogonadism. Further investigations are warranted for the differential diagnosis of congenital and acquired hypothalamic–pituitary disorders, also taking into account data from medical history and physical examination. In these patients, measurement of prolactin (PRL) levels is justified to reveal or exclude the presence of a PRL-secreting pituitary adenoma.
- Low testosterone with elevated serum gonadotrophins is indicative of hypergonadotropic hypogonadism. In Klinefelter syndrome, this endocrine profile is associated to very small (< 5 mL) and firm testes; karyotype analysis confirms the diagnosis [19].
- Elevated serum FSH levels with normal LH and testosterone suggests an isolated primary spermatogenetic failure, representing the most common cause of NOA. In these patients, testis volume is often, but not necessarily, reduced. Specific etiologies include orchitis, spermatogenesis damage due to chemo-/radiotherapy, cryptorchidism (especially when bilateral and/or subjected to late treatment), and Y chromosome microdeletions. Unfortunately, causes remain unknown in more than 50% of cases.
- Normal serum levels of FSH, LH and testosterone associated to normal testicular volume imply the need for a cytological/histological differential diagnosis between OA and isolated primary spermatogenetic failure. In fact, serum FSH levels may be normal in case of azoospermia due to postmeiotic spermatogenic arrest [20].

Obstructions may occur at any level of the male genital tract from the epididymis to the ejaculatory duct, but only complete and bilateral obstructions can result in OA. Apart from acquired causes, including inflammatory, traumatic and iatrogenic aetiologies (e.g. inguinal/scrotal surgery, vasectomy), specific genetic defects could be involved in OA, leading to congenital malformations of male genital tract. In particular, congenital bilateral absence of the vas deferens (CBAVD) represents a minor variant of cystic fibrosis (CF) and results from mutations in the CF transmembrane conductance regulator (CFTR) gene [21, 22]. In the presence of azoospermia, some seminal features can orient towards obstructive disorders. The alkaline secretion of seminal vesicles accounts for the bulk volume of the ejaculate; it is rich in fructose and contains semenogelins, which are involved in seminal coagulation. Therefore, the combination of hypospermia, acid pH of the ejaculate, low seminal fructose concentration and absence of seminal clot is highly suggestive of OA due to CBAVD or bilateral obstruction of ejaculatory ducts. In patients with OA, scrotal US can document enlargements in rete testis and epididymis [12, 23]; in CBAVD, seminal vesicles are not detectable at the transrectal US [12].

### Diagnostic work up of OAT

A variable combination of low sperm count, poor progressive motility and poor sperm morphology is frequently found in infertile men. The actual aetiology of OAT remains unknown in many cases, after excluding several possible contributing factors, including varicocele, orchitis, and urogenital infections, cryptorchidism, testicular injuries, systemic diseases and fever, immunosuppressive treatments, chemo-/radiotherapy, exposure to toxicants and physical agents, and anabolic substance abuse [14]. In the presence of urogenital infections, semen analysis might document leukocytospermia (> 1 million leukocytes/mL) with increased viscosity and pH of the ejaculate. In the likelihood of a urogenital infection, sperm culture with antibiotic sensitivity testing is recommended.

Men with OAT should routinely be offered endocrine evaluation of FSH, LH and total testosterone levels for diagnosis of hypogonadism, given the increased risk of androgen deficiency in men with impaired semen quality [14]. Determination of PRL should be also included if a hypogonadotropic hypogonadism is suspected.

Scrotal US should be regarded as an integral part of routine investigations of men with OAT [14]. Gray-scale sonograms allow the accurate assessment of testicular volume and texture, the evaluation of epididymis size and texture and the detection of enlargements in the pampiniform venous plexus [11, 12]. When combined with colour Doppler spectrum analysis, US also provides a quantitative measure of spermatic venous reflux in patients with clinical varicocele [24, 25] and can identify subclinical varicocele or assist in the follow-up of varicocele repair [26]. Of note, infertile oligozoospermic men exhibit an increased risk of testicular germ cell tumour (TGCT) compared with fertile control subjects [27] and, in infertile men, the presence of testicular microlithiasis is associated to an about 18-fold higher prevalence of testicular cancer [28], thus further supporting the recommendation to perform scrotal US in all infertile men with OAT [14].

As for genetic tests, it should be considered that severe OAT can be associated with autosome translocations that potentially increase the risk for an unbalanced karyotype in embryos [29]. It is worth stressing that due to multi-faceted interactions between karyotype abnormalities, general and reproductive health, a careful evaluation and a multidisciplinary approach in this setting is advisable [30–32].

Furthermore, Yq microdeletions (which are transmitted to the male embryo), albeit rarely, might be a cause of severe oligozoospermia/cryptozoospermia [29]. Therefore, karyotype analysis and assessment of Yq microdeletions are recommended in infertile men with a sperm concentration ≤ 5 × 10^6^/mL [14].

Finally, the addition of a sperm DNA integrity testing to standard semen analysis, albeit still debated, can provide further information on the couple’s chance of spontaneous pregnancy and in selection of method of assisted reproduction [14, 33, 34]. These issues will be further discussed in the following paragraphs.

## Seminology and assisted reproduction

As stated before, semen analysis is indeed the first level examination in the evaluation of male factor infertility [35]. It is, thus, imperative to perform such analyses in highly specialized centers, where trained seminologists follow the latest WHO recommendations [13]. Both a macroscopical and a microscopical evaluation should be performed. The latter in particular should evaluate concentration, motility and morphology of spermatozoa and the presence of other cellular components (round cells such as leukocytes, spermatogonia, spermatids and other cells: epithelial cells and red cells). Oligozoospermia, asthenozoospermia and teratozoospermia are defined by sperm parameters below the WHO 2010 5th percentile (that is a total sperm count below 39 × 10^6^, progressive motility below 32% and abnormal forms above 96%). It is worth noting that WHO 2021 does not propose precise “pathological” thresholds due to a strong overlap between fertile and infertile sperm parameters and, therefore, suggests the clinicians to interpret sperm parameters within the broader clinical context [13, 36].

For the andrologist, the first necessary step is the interpretation of these sperm parameters in light of the available clinical information. A correct clinical classification will allow the clinician to optimize the therapeutic strategy also to determine the type of assisted fertilization technique to use. Indeed, first level should apply in the case of normozoospermia, while second and third levels, more elaborate and expensive, should be reserved in case of severe male factor with heavy alteration of sperm parameters.

In the presence of a confirmed azoospermia, as well as in case of severely reduced semen volume and/or whenever a retrograde ejaculation is suspected (prostate or bladder neck surgery, neuropathy, etc.), the presence of spermatozoa should also be investigated in a post masturbation urine sample. In case spermatozoa are found, urine alkalinisation (through diet and/or intake of sodium bicarbonate) might allow to cryopreserve these cells or to directly use them in ART [37]. However, most of the studies reporting successful isolation of sperm from urine are relatively small case series, with a per-cycle pregnancy rate ranging from 20 to 50% [37].

Wide intra-individual variability of sperm parameters presents a real challenge for the clinical andrologists, as it may also be influenced by a variety of factors (incomplete collection, fever, drugs, such as antibiotics, etc.) that must be investigated during medical history collection. Possible centre by centre and technical problems should be considered [38]. Furthermore, at least one repetition of semen analysis is necessary to confirm its result, especially in the presence of alterations, in order to define the need of a specific treatment, including ART.

Another parameter that should be considered before ART is the viability of the spermatozoa. Its evaluation is rather easy, and it is currently recommended in the case of samples with reduced motility [13]. The functional integrity of the spermatozoa is evaluated using dyes that do not pass through an intact membrane of a viable cell. This is particularly important in the era of assisted fertilization, as it allows to estimate semen samples potentially capable of fertilizing oocytes.

Knowledge of sperm selection techniques is also important for the andrologist as these select the best spermatozoa and optimize fertilization rates, representing a necessary step to choose the assisted fertilization technique to be used.

Instead, in case of idiopathic infertility (especially if both sperm agglutination and reduced motility are present), the presence of antisperm antibodies may be investigated. Blood–testis barrier defends male gametes from the immune system, but several conditions (inflammation, traumas, testicular torsions, cryptorchidism, vasectomy, etc.) could impact in its integrity, potentially leading to an autoimmune response [39, 40]. In general, antisperm antibodies are known to potentially interfere with reproduction through different mechanisms affecting sperm fertilization capabilities [41]. Antisperm antibodies are present in 4 to 10% of unselected men attending a fertility clinic [42, 43].

ART is considered the elective treatment in case of ASA, with no significant difference in the reproductive outcome of IVF and ICSI reported in their presence [40]. IUI may be also a valid first level option, favoring fertilization bypassing the obstacle of cervical mucus [44]. However, high levels of antibodies may interfere with spermatozoa interaction with oocyte membranes, significantly reducing fertilization rates [45].

In conclusion, as it has been made clear, the clinical andrologist should use the expertise and experience in interpreting semen parameters not only to investigate and treat male factors underlying seminological alterations [46–48], but also to assist other reproductive medicine specialists in the choice of the proper-assisted reproduction technique.

## Fertility preservation and ART—oncofertility

Sperm and testicular tissue cryopreservation are widely used techniques to maintain reproductive cells and tissues in a vital state through the use of cryogenic temperatures and cryoprotectants. This allows to prevent freezing damage to male gametes that may potentially be successfully used in ART even after many years. In fact, andrologists working in a Sperm Bank aim to both to preserve patients’ fertility and to facilitate the access to ART. Potential indications to sperm cryopreservation are many, including cancer treatments, autoimmune and urological diseases. In general, the andrologist working alone or in a multidisciplinary team should recommend the access to a fertility preservation service whenever a patient faces a condition or treatment that might interfere either with spermatogenesis and genome integrity or ejaculation mechanisms [49–51]. It is obvious that patients diagnosed with cancers in the reproductive age (mostly testicular cancers and lymphomas) are the primary recipients for sperm cryopreservation [52–56]. Furthermore, the andrologist may suggest sperm cryopreservation whenever it may facilitate ART procedures, such as the cases of patients with spinal cord injury [57, 58] and of those with severe alteration of spermatogenesis risking high fluctuations of semen quality [59]. In the latter case, in the presence of severe OAT, fluctuations may result in azoospermia, either transient or permanent. Consequently, a previously cryopreserved semen sample may avoid wasting an ovarian stimulation in the female partner. Patients with azoospermia, as those with Klinefelter syndrome, may benefit from cryopreservation after testicular sperm extraction (TESE)/microTESE [60, 61]. Testicular biopsy is a relatively safe procedure and spermatozoa can be retrieved in about 50% of cases [62]; timing of TESE/microTESE should be evaluated on the basis of clinical and hormone variables (age, testicular volume, endogenous FSH levels, etc.) [63]. Regardless of the specific case or procedure, before accessing to fertility preservation and ART, the andrologist should screen the patient for the presence of several viruses (HBV, HCV, CMV, HIV, among others). In fact, viruses possibly present in the semen sample and cryostored in liquid nitrogen are able to maintain their pathogenic properties [64]. Some viruses, can be isolated from the seminal fluid of infected men, as local testicular inflammation might render the blood–testis barrier permeable to viruses [65]. Viruses like Zika may show long-term persistence in the seminal fluid, with possible negative effects on ART [66]. Also, the recent SARS-CoV-2 pandemic has caused relevant concerns for possible consequences of coronavirus infection on assisted reproduction. Although there is still limited evidence, recent papers seem to generally agree on the absence of SARS-CoV-2 in semen [67] and a report from a small caseload on asymptomatic patients undergoing sperm cryopreservation suggests that SARS-CoV-2 may also not be detected in cryopreserved samples [68]. Consequently, chances of viral transmission from semen samples during assisted reproductive techniques seems unlikely.

Fertility preservation counselling, also in an oncofertility setting, should be regarded as one of the challenges that the andrologists face in their clinical practice. Cancer patients in fertile age may also find in sperm cryopreservation a strong psychological support to deal with the various stages of treatment protocols [69]. Patients preserving their fertility before cancer treatment should be counselled regarding the future of their samples and the chances of recovery of natural fertility. Previous reports show that various degrees of damage to spermatogenesis (up to azoospermia) may transiently or permanently affect the patient after antineoplastic treatments. Azoospermia in particular can be present in up to 3–6% of patients in testicular cancer patients two years after chemo- or radiotherapy [52], partly depending on the treatment type and dose [54]. Hematological cancers, that may require more intensive treatments may cause a higher incidence of azoospermia or permanent alteration of spermatogenesis in the same time frame [56]. Nonetheless, patients should be informed that even in case of permanent damage to fertility, the use of cryopreserved semen samples is reported to have cumulative rates of fatherhood close to 50% [49]. Thus, the discussion of post-treatment fertility and possible use of ART, as well as sperm cryopreservation strategies, should be encouraged.

Furthermore, the setting of oncofertility puts the patient in an integrated pathway where the andrologist works in tandem with the oncologists and other relevant specialists (seminologist, infectious diseases, urology, psychology, bioethics, etc.) to fulfil the oncological patient needs. The andrologist, in particular, supported by the seminologist, should offer fertility preservation counselling and a careful follow up of testicular function evaluating semen quality, hormonal profile and testicular US. He/she should also guarantee proper treatment to the patient once the desire for natural fertility arises. Also, he/she should discuss with the patient about the chance to use the cryopreserved semen in ART.

In conclusion, the role of the andrologist in fertility preservation should not be solely seen as the chance to offer a fertility-oriented discussion (either towards ART or natural fertility), but also as the chance to accompany the oncological patients offering appropriate screening and treatment both at diagnosis and during the entire follow-up until the patients’ reproductive need is fulfilled.

## Sperm DNA fragmentation in male infertility

Semen analysis represents only one side of the interpretation of sperm parameters. There is an increasing awareness that “qualitative” markers such as sperm chromatin and DNA integrity and oxidative stress are also essential to evaluate the ability to fertilize and for the subsequent normal development of the embryo [70]. The wide overlap between sperm parameters of fertile and infertile men, have increased the demand of a diagnostic test capable of investigating the male reproductive capability both at the diagnostic and at the therapeutic stage (for example, after a treatment and/or before ART) [71]. During spermiogenesis a major reorganization of the genome occurs, in parallel to radical morphological changes of the male gamete. Sperm DNA strand breaks may occur physiologically during spermiogenesis but the action of both endogenous and environmental factors may increase these breaks and cause unrepairable damage [55]. An excessive production of oxygen free radicals or an apoptotic process may induce sperm DNA damage. Ageing and environmental stress factors inducing oxidative stress, genetic mutations and chromosome abnormalities can cause protamination defects with negative repercussions on chromatin structure and fertility [72].

Sperm DNA integrity has risen in importance in light of the widespread use of ART as an increasing amount of evidence is associating it to adverse reproductive outcomes, recurrent pregnancy loss and reduced pregnancy rate, both in natural cycles and in ART: sperm DNA damage has been associated with a lower chance and longer time to achieve a pregnancy through natural fertility [73, 74]; likewise, SDF seems to be associated with lower pregnancy rates after ART and with higher chance of worse outcomes (pregnancy loss in particular) after either IVF or ICSI [75–77]. A clear association has been also detected between SDF and recurrent pregnancy loss [78].

The clinical andrologist should also be aware that the detection method may highlight slightly different associations since different forms of DNA damage are identified. TUNEL is a relatively common method, which is based on the use the enzyme terminal deoxynucleotide transferase (TdT), which catalyses the polymerization of fluorescein-labelled nucleotides to the 3′-OH terminal end of the fragmented DNA [79]. SCSA and Comet assay are also frequently used [55]. Alkaline Comet and TUNEL assays are direct methods as they give back a direct measure of sperm DNA damage, while measures from SCSA indicate a susceptibility of DNA to damage, ultimately influencing the associations with reproductive outcomes. Most notably, studies using TUNEL tend to concur about a significant impact on fertilization outcomes and pregnancy loss for both IVF and ICSI, while studies conducted using SCSA showed less constant results [77, 80, 81]. Despite many possible applications for sperm DNA fragmentation testing, it should be remembered that the final effect on reproduction of sperm DNA damage is not only function of the percentage of sperm with fragmented DNA, but also of the oocyte DNA repair capabilities. In fact, the net biological effects of an abnormal chromatin structure may depend on the combination of both severity of sperm chromatin damage and oocyte quality.

In clinical practice, many scientific societies still do not recommend routine testing for sperm DNA fragmentation. Available evidence seems to limit the value of sperm DNA damage evaluation in specific settings where the andrologist may provide counselling to the infertile couple before referring to assisted reproduction. Furthermore, sperm DNA damage evaluation may allow the identification of specific subsets of patients at risk for recurrent pregnancy loss [35, 82–84].

## Therapeutic management of the infertile man

The management of the infertile male does not reach its completion after a diagnosis is made. Once the andrologist identifies a specific disease, it is his duty to guide the patient to the etiological treatment, either surgical (for example, varicocele repair) or medical, and to present to the patients (or better, to the couple) the expected benefits of the treatment as well as to set up the appropriate follow-up. Also, in a number of couples a specific aetiology cannot be detected but in selected cases of idiopathic infertility a treatment can also be proposed. It should be stressed that smoking, dietary habits, sedentariness, drug abuse, professional expositions have been proposed as factors capable to affect ROS production and possibly increasing oxidative stress in semen with consequent alteration of sperm parameters and worsened sperm DNA fragmentation [85]. The detrimental potential of these lifestyle factors on male fertility is still largely speculative and further studies are needed to affirm this relationship [85]. Moreover, studies evaluating change in lifestyle are lacking. Obesity is among the most widely studied factors. The relationship between obesity and male infertility is debated and, at best, mild [86]. Accordingly, a meta-analysis of 28 cohort studies, involving 1022 obese men undergoing bariatric surgery, failed to show any improvement in semen parameters after weight loss [87]. Nonetheless, correction of these wrong lifestyles should be suggested before medical treatments in the “arsenal” of the andrologist, which is composed of “hormonal” and “non hormonal” treatments (Table 1). On the other hand, the intrusion of medical prescriptions and treatments (including ART) may be negatively perceived by the infertile couple, increasing levels of perceived stress and affecting the couple’s quality of life with repercussion on their sexual health. This aspect, sometimes neglected, should be considered in the context of any fertility treatment [88].

**Table 1:** Summary of main available treatments for the infertile men

Treatment	Level of evidence	Advantages/disadvanteges	References
Hormone treatment (hypogonadotropic hypogonadism)			
GnRH or gonadotropins	High	Pros Efficacy in improving semen parameters and pregnancy rate Cons Costs Subcutaneous injection delivered by a pump that must be worn 24 h/day Feasible only in men with functional pituitary gland	Jungwirth et al. [89], Cassatella et al. [91], Rastrelli et al. [86], Gong et al. [92], Mao et al. [93], Lin et al. [94]
FSH ± human chorionic gonadotropin (hCG)	High	Pros Efficacy in improving semen parameters and pregnancy rate Cons Costs Intramuscular or subcutaneous injections weekly	Howard and Dunkel [95], Rastrelli et al. [86], Nieschlag et al. [97]
Hormone treatment (idiopathic infertility)			
FSH	Medium–Low	Pros Improved pregnancy rate Cons Costs Few clinical trials dosage “imported” from treatment of HH	Barbonetti et al. [98], Simoni et al. [99], Attia et al. [100], Santi et al. [101], Paradisi et al. [102], Ding et al. [103], Santi et al. [104]
Hormone treatment (idiopathic infertility)			
Selective estrogen receptor modulators (SERMs) and aromatase inhibitors	Low	Pros Few adverse effects (short term) Low costs Cons Off label Limited evidence available (not recommended by available guidelines)	Vandekerckhove et al. [105], Chua et al. [106], and Del Giudice et al. [107]
Non-hormone treatment (idiopathic infertility)			
Antioxidants and nutraceuticals (not recommended by available guidelines)	Low	Pros Widespread Cons Risk of excessive self-medication without medical supervision inconclusive evidence available (not recommended by available guidelines)	Smits et al. [109] and Lombardo et al. [108]

### Hormone treatment for infertile men

- GnRH or gonadotropins

In secondary hypogonadal men (hypogonadotropic hypogonadism), gonadotropin releasing hormone (GnRH) or gonadotropin replacement is a rationale treatment that demonstrated efficacy in improving semen parameters and pregnancy rate. Accordingly, they are strongly recommended by the guidelines on male infertility [89]. GnRH is less and less used because its administration is cumbersome, relying on subcutaneous injection of GnRH delivered by a pump that must be worn 24 h/day. The pump releases 100–400 ng/kg of GnRH with pulses every 90–120 min mimicking the physiological GnRH secretion pattern [90]. Obviously, this treatment is feasible only in men with intact pituitary and normal functioning GnRH receptors thus excluding those with pituitary diseases or normo-osmic hypogonadotropic hypogonadism for GnRH receptor mutations [91]. The meta-analysis of the studies, which used GnRH treatment in azoospermic secondary hypogonadal men showed that this is an effective treatment inducing the appearance of sperms in the ejaculate in 75% of cases on average with a mean sperm concentration of 4.3 × 10^6^/mL [86]. These results were comparable or slightly worse than those obtained in subjects treated with gonadotropins [86] but few head-to-head comparison studies reported shorter time required with GnRH to achieve spermatozoa in the ejaculate [92–94].

Nowadays, gonadotropins are more conveniently used because their administration requires three intramuscular or subcutaneous injections weekly. The optimal dose and schedule for this treatment is not agreed and possible regimens encompass the use of human chorionic gonadotropin (hCG) alone or hCG together with FSH-like preparations. hCG dose varies 1500–3000 IU twice weekly with titration according to serum testosterone levels, whereas FSH-like preparations are administered 75–150 IU two or three times per week [95]. A pre-treatment with FSH alone has been hypothesized in congenital hypogonadotropic hypogonadism [96], but it currently has no role in the management of idiopathic infertility treatment. A meta-analysis of the available studies has clearly shown that combined therapy with hCG and FSH-like preparations is significantly more effective than hCG alone allowing the attainment of sperm output in 80% of previously azoospermic men vs. 50% found in hCG treated men, with a mean sperm concentration of 12 vs. 1 million/mL [86]. Although semen concentration remained overall below the lower limit of normality (5.9 [4.7;7.1] × 10^6^/mL), pregnancy was observed in 50% of cases and the pregnancy rate may be even higher considering that not all subjects included in the primary studies were seeking fertility [86].

Recently, a new compound has been introduced for the treatment of secondary hypogonadal men seeking fertility. This is corifollitropin alfa, a recombinant gonadotropin made of the α-subunit of human FSH and a hybrid part composed of the β-subunit of human FSH and the carboxy-terminal of the β-subunit of hCG. Corifollitropin alfa has longer half-life and time to achieve peak levels in the blood. This allows administration every other week thus the compliance may be improved. At present, corifollitropin is marketed for female infertility. A pre-marketing study for adult male treatment has shown that 77.8% of azoospermic men with secondary hypogonadism treated with hCG twice weekly and corifollitropin 150 mcg every other week for 52 weeks achieved ≥ 1 million of sperm/mL with a final mean sperm count of 5.2 × 10^6^/mL [97].

- FSH use in idiopathic male infertility

Gonadotropin treatment is also commonly used for idiopathic male infertility [98]. In this case, treatment is based on the administration of FSH despite a clear gonadotropin deficiency is not documentable. For this reason, FSH treatment in idiopathic infertile men is often referred to as an empirical therapy. However, it relies on the rationale that, in OAT men, low-to-normal FSH levels are actually inappropriate thus denoting an insufficient stimulation of the spermatic epithelium. Similar to secondary hypogonadal men, the schedule and dose for FSH treatment in idiopathic infertile men with altered semen parameters is not agreed. Most randomized clinical trials (RCTs) use doses varying 50–300 IU administered daily or every other day [99]. The effectiveness of FSH therapy in idiopathic male infertility is debated because clinical trials and meta-analyses led to conflicting results. In 2013, the Cochrane Collaboration published a meta-analysis on the use of FSH in males with idiopathic infertility, which accrued data from 6 RCTs with overall 456 participants [100]. The meta-analysis showed a significant fivefold increase in spontaneous pregnancy rate associated to FSH treatment but no change in pregnancy rate after assisted reproduction techniques (ART) or live-birth rate [100]. However, only one study participated to the estimation of the latter outcomes, thus limiting much the conclusions that could be drawn. The strictness of the methodological approach of the Cochrane Collaboration, despite favouring the homogeneity of the included trials, limits strongly the completeness and the up-to-datedness of the information. The inclusion of all controlled trials independent of the randomization and the kind of control arm (placebo or untreated) allowed to include, in a following meta-analysis, 15 trials involving 1275 infertile men [101]. This study showed an increase in sperm concentration of 2.7 [0.5; 4.8] × 10^6^/mL that was statistically significant, whereas progressive sperm motility was only slightly and not significantly improved (1.2% [− 0.1; 2.5]). In addition, the meta-analysis confirmed the higher spontaneous pregnancy rate in couples where the male partner received FSH therapy and also found a significantly increased success of ART [101].

The inconsistent results reported by trials on FSH treatment in idiopathic male patients may find different explanations. First, the treatment was initially translated directly from secondary hypogonadism and similar dosages were applied. It is conceivable that these are not sufficient, as suggested by higher sperm output obtained when higher dosages are applied [102, 103]. Besides optimizing the dosage, a standardization of the treatment duration is required. Trials published so far mainly reported results after 12 weeks of treatment [99, 104]. However, it is conceivable that longer treatments are required, at least covering two spermatogenetic cycles (approximately six months) to ensure the best results.

- Antiestrogens

Selective Estrogen Receptor Modulators (SERMs) and aromatase inhibitors have been used for the treatment of male infertility by leveraging their property to inhibit the negative feedback on the hypothalamic–pituitary played by the estrogens. This results in increased gonadotropin release with greater testis stimulation of either the spermatogenetic or the steroidogenetic compartment. SERMs and aromatase inhibitors are used in infertile men without a clear hypogonadism due to gonadotropin deficiency and they produce an overstimulation of FSH-mediated mechanisms and increased testosterone concentration within the testis.

The efficacy of SERMs in idiopathic male infertility has been initially quantified the Cochrane Collaboration [105], which found, in placebo controlled RCTs, a small non-statistically significant increase in pregnancy rate and concluded that there is not enough evidence to draw conclusions on SERMs efficacy in this clinical setting. More recently, a meta-analysis of 11 placebo-controlled or open-label RCTs [106] has shown that subjects taking SERMs achieved a significant increase in sperm concentration (5.2 [2.1; 8.4] × 10^6^/mL) and motility (4.6 [0.7; 8.4] %) without any significant change in normal morphology (− 0.3 [− 0.6; 0.1] %). Besides this relatively modest improvement in semen parameters, treatment with SERMs was associated with a two-fold increase in pregnancy rate that, however, is obtained only with higher dosages of tamoxifen (20–30 mg daily) or clomiphene (50 mg daily) [106].

Recently, a meta-analysis of eight studies has found a significant increase in sperm concentration (2.6 [1.8; 3.4] × 10^6^/mL) and motility (2.3 [1.1; 3.5] %) in infertile men after treatment with aromatase inhibitors [107]. Unfortunately, the small number of available trials and the mixed study design (randomized clinical trials and longitudinal prospective and retrospective studies) strongly limits this meta-analysis. In addition, data on pregnancy rate or other measures of fertility outcomes were not available from the studies included.

Despite the treatment with SERMs or aromatase inhibitors in infertile men is quite popular particularly in some regions, available evidence is very limited and no conclusions could be drawn on their efficacy. Accordingly, available guidelines [14, 89] do not recommend their use. While recognizing that they have few adverse effects, particularly for short-term therapy, and relatively low costs, it should be underlined that they are off-label treatment in most countries (including Italy).

### Nonhormone treatment for infertile men

- Antioxidants and nutraceuticals

Oxidative stress is deemed the molecular mechanism contributing to male subfertility in a number of clinical conditions. Exogenous (environmental or lifestyle) and endogenous (i.e. infections, chronic diseases, varicocele) factors potentially induce an increased production of reactive oxygen species (ROS) with possible adverse effects on fertility [108]. According to this hypothesis, several antioxidants or dietary supplements with antioxidant properties, alone or combined, have been studied in clinical trials with mixed results. Nonetheless, the use of these compounds has spread beyond the scientific evidence of their efficacy. The reasons of their attractiveness towards either males from infertile couples or physicians is that nutraceuticals are relatively inexpensive and devoid of adverse events. They can be purchased in several contexts from pharmacies to supermarkets or fitness centers without any medical supervision. This could lead to excessive self-medication with the risk of missing possible diagnosis and/or treatments with proven efficacy. Indeed, antioxidants are still not recommended treatments for male infertility due to the limited evidence on their efficacy. A recent update of a meta-analysis on this topic by the Cochrane Collaboration [109], while highlighting the scarce quality of the available studies, has shown a positive effect on live births and clinical pregnancy rate of antioxidants as compared with placebo or no treatment [OR = 1.8 [1.2; 2.7] and 2.9 [1.9; 4.6], respectively] [109]. The small number of studies available for each compound limited the possibility to evaluate if there is a specific antioxidant or combination of antioxidants, which provide better results. Overall, the poor quality of evidence does not allow drawing conclusion and, pending larger, well-designed and adequately powered RCTs, available guidelines do not recommend the use of antioxidants in males from infertile couples [14, 89]. However, the use of nutraceuticals could be reasonable in subjects that, after a thorough andrological evaluation, resulted not to have conditions causing infertility–- therefore classified as idiopathic infertile–- when other therapies with higher level of evidence (i.e. FSH) were unsuccessful [110].

## Future perspectives in the male infertility treatment

Future perspectives in the clinical management of male infertility would consider all challenges still present in treating men with infertility due to either HH or idiopathic. In these clinical scenarios, the appropriate diagnostic–therapeutic framework remains still partially unknown. In the setting of HH, it is largely demonstrated that fertility could be restored with gonadotropins administration. However, the most effective therapeutic scheme in terms of sperm parameters improvement and pregnancy rate is still far to be identified. Despite a physiological spermatogenesis requires a synergic action of LH and FSH, several authors highlighted a sperm number increase in HH men using only hCG [86, 89, 111]. Thus, is it conceivable that the FSH action is redundant? Some evidence suggested that the FSH addition in HH men induces a more pronounced increase in sperm number and a globally increased sperm quality, compared to the luteinizing action alone. Therefore, if on one hand the action of FSH seems to optimize the restoration of spermatogenesis, on the other hand, the optimal time to introduce the therapy as well as the most suitable therapeutic scheme are actually proposed on an empirical basis. With this in mind, it is clear that proper designed clinical trials are needed to identify the optimal FSH administration timing and dosages. Another point of debate is represented by the comparison of the luteinizing action induced by hCG and LH in men. Indeed, in light of its easier obtainability, hCG is still used to stimulate intratesticular testosterone raise instead of LH. From a physiological point of view, although hCG and LH bind the same membrane receptor, their action is proven to be different both at molecular level [112] and in women undergoing assisted reproduction [113]. Nevertheless, hCG is preferred to LH for historical and practical reasons, due to the higher availability, the longer half-life and the relative low cost [114]. Recently, recombinant techniques allowed the production and distribution of new LH compounds that could be tested in male infertility setting. Nowadays, it is largely demonstrated that the hCG + FSH administration to HH men stimulates spermatogenesis, but without restoring it up to normozoospermia [115]. Thus, the combined gonadotropin administration increases the semen quality, rather than its quantity. Perhaps the restoration of the physiological gonadotropic stimulus on the testis (i.e. LH + FSH) could completely re-establish sperm production in HH men. This intriguing hypothesis remains to be proved by future prospective interventional trials aiming to detect the best therapeutic option to completely restore spermatogenesis in HH men. In the setting of male idiopathic infertility, the future perspectives are even broader, since there are still many obscure points in the present therapeutic management. Currently, exogenous FSH administration could be proposed in the setting of male idiopathic infertility, in accordance with regulations that drastically differ between different countries [104]. Indeed, the theoretical testicular overstimulation with exogenous gonadotropins is still empirical [104]. Moreover, the absence of an etiological diagnosis, i.e. the definition of idiopathic infertility, interfere—if not entirely preclude—the detection of a clinically efficient treatment. With these limiting premises, a pharmacogenomics approach could help at identifying which patients could be effectively treated with exogenous gonadotropins stimulation and how tailor the therapeutic scheme on each patient. Pharmacogenomics starts from the demonstration that FSH action, both in physiological and therapeutic conditions, could be influenced by the presence of single nucleotide polymorphisms (SNPs) on the FSHR (c.919A > G, rs6165; c.2039A > G, rs6166; -29G > A, rs1394205) and the FSHB genes (-211G > T, rs10835638) [116–118]. Since the first description of the potential role of FSHR SNPs on human reproduction [119], this topic has been extensively studied in women undergoing assisted reproduction, trying to optimize therapeutic schemes. The overall evaluation of the pharmacogenomics role in women infertility supports the relevance of specific FSHR/FSHB genotypes, on the basis of which the controlled ovarian stimulation phase can be customized [120]. However, considering the male counterpart, the modulatory activity exerted by the FSHR c.2039A > G SNP was demonstrated only in 2012 [121, 122]. Men carriers of the homozygous G variant showed lower testicular volume and higher FSH serum levels compared to homozygous A or heterozygous A/G patients [121, 122]. Similarly, the FSHB c.-211G > T was demonstrated to influence male fertility, since T homozygosity resulted associated with lower testicular volume, sperm count, testosterone, and LH serum levels [122]. Recently, Wu et al. highlighted a specific haplotype more frequent in fertile men considering the possible combinations of the three FSHR SNPs (i.e. c.919A > G A allele, c.2039A > G A allele and -29G > A G allele) [123]. Thus, a combined effect of FSHR and FSHB SNPs variants should be considered to better understand the potential of the pharmacogenomic approach. So far, the first, and still unique, study with a proper pharmacogenomic design in idiopathic male infertility has been published in 2016 [124]. In this clinical trial, 66 men with idiopathic infertility were treated with FSH 150 IU every other day for three months, showing a significant increase in semen quality (in terms of sperm DNA fragmentation reduction) only in men with FSHR c.2039A > G A homozygous and FSHB -211G > T G homozygous [124]. Thus, a specific genetic haplotype seems to predict the response to FSH stimulation. Although the literature is still poor in studies confirming the pharmacogenomics role in assessing the FSH administration efficacy, this approach could be useful for a priori selection of patients who will potentially benefit from FSH treatment. However, other pharmacogenomics studies are needed to prospectively evaluate how to personalize FSH treatment according to FSHR/FSHB genotypes. On the other hand, it is conceivable that those haplotypes associated with a worse fertility phenotype could benefit of higher FSH dosages or longer treatment duration. Thus, future perspectives should combine the genetic background to the treatment response, also in a cost–benefit perspective. Finally, future perspectives in male infertility treatment should consider new compounds, such as corifollitropin alpha (see above). Next to corifollitropin alpha, other compounds have been developed to mimic the action of gonadotropic stimulus, such as (i) single-chain gonadotropins and (ii) low molecular weight chemicals acting as FSHR agonists. Single-chain gonadotropins present improved pharmacokinetics [125], increased in vivo bio-potency [126] and longer half life [127–129]. Conversely, FSHR agonists have the relevant advantage of the oral route of administration [130]. Although all these compounds are currently evaluated in experimental models, no attempts have been performed so far in clinical practice. Thus, future perspective must consider these innovative compounds to develop new therapeutic strategies able to improve the efficacy and the compliance of infertility treatments.

## Conclusions

In conclusion, the current role of the andrologist poses himself in the middle of a complex decisional network, where benefits and costs of the diagnostic and therapeutic work up must be carefully balanced in order to adequately support the infertile couple. Thus, the andrologist’s clinical setting, in order to provide a high-level of care, must include several critical diagnostical and therapeutical steps. Even though ART may be the final and decisive stage of this decisional network, neglecting to treat the male partner may ultimately increase the risks of negative outcome, as well as costs and psychological burden for the couple itself.
